# The Role of Ultrasonography in Predicting Genetic Characteristics of Endometrial Cancers

**DOI:** 10.3390/jcm14093216

**Published:** 2025-05-06

**Authors:** Kemine Uzel, Filiz Bilir, Mesude Tosun, Nura Fitnat Topbas Selcuki, Seda Eren Keskin, Merve Gokbayrak, Gulhan Demir, Naci Cine, Pasa Ulug, Ahmet Cem Iyibozkurt, Hakan Savlı

**Affiliations:** 1 Department of Obstetrics and Gynecology, Demiroglu Science University, 41100 Istanbul, Turkey; cemiyi@yahoo.com; 2 Department of Obstetrics and Gynecology, Faculty of Medicine, Kocaeli University, 41001 Kocaeli, Turkey; drflzyldz@hotmail.com; 3 Department of Radiology, Faculty of Medicine, Kocaeli University, 41001 Kocaeli, Turkey; mesudetosun@hotmail.com; 4 Department of Obstetrics and Gynecology, Istanbul Sisli Hamidiye Etfal Training and Research Hospital, University of Health Sciences Turkey, 34000 Istanbul, Turkey; fitnat.topbas@gmail.com; 5 Department of Genetics, Faculty of Medicine, Kocaeli University, 41001 Kocaeli, Turkey; sedaerenkeskin@gmail.com (S.E.K.); merve_ertan09@hotmail.com (M.G.); gulhand93@gmail.com (G.D.); nacicine@yahoo.com (N.C.); hakansavli@yahoo.com (H.S.); 6 Department of Obstetrics and Gynecology, Istanbul Haseki Training and Research Hospital, 34096 Istanbul, Turkey; pasaulug@hotmail.com

**Keywords:** elastography, endometrium cancer, cell-free tumor DNA (ctDNA), tumor stiffness

## Abstract

**Background/Objectives:** To evaluate the association between endometrial tissue stiffness, as measured by shear wave elastography (SWE), and the presence of specific gene mutations in patients diagnosed with endometrial cancer. **Methods:** Peripheral blood samples were collected for DNA extraction and next-generation sequencing (NGS) to identify gene mutations. Preoperative SWE was performed to measure endometrial stiffness, with values expressed in kilopascals (kPa). Statistical analyses were conducted to assess the correlation between SWE measurements and genetic findings. **Results:** Genetic mutations were detected in 66% (n = 31) of cases, with TTN, PLEC, and PRSS1 being the most frequently mutated genes. The median SWE measurement was 36.5 kPa (range: 19.1–70.4 kPa). No statistically significant correlation was found between SWE values and the presence of gene mutations (*p* > 0.05). Cases with metastasis exhibited higher median SWE values (40.1 kPa) compared to non-metastatic cases (34.7 kPa), though this difference was not statistically significant. **Conclusions:** While no significant association was observed between endometrial stiffness and specific gene mutations, higher SWE values in metastatic cases suggest that increased tissue stiffness may be linked to tumor aggressiveness. Further large-scale studies are warranted to validate these findings and explore the potential of SWE as a non-invasive tool in assessing endometrial cancer characteristics.

## 1. Introduction

Endometrial cancer is the most prevalent gynecological malignancy in developed nations and ranks as the sixth most frequently diagnosed cancer worldwide. Several risk factors contribute to its development, including advanced age, obesity, unopposed estrogen exposure, diabetes, and hereditary nonpolyposis colorectal cancer [1]. Histologically, endometrial cancer is broadly classified into two main subtypes: type I and type II. Type I, the more common subtype, is characterized by endometrioid histology and is associated with mutations in the PTEN, KRAS, CTNNB1, and PIK3CA genes, along with hormone receptor expression [2,3,4,5]. In contrast, type II tumors exhibit non-endometrioid histology, are frequently associated with high-grade features, and demonstrate p53 and p16 alterations, HER-2 overexpression, and a lack of hormone receptor expression [6,7,8].

TTN mutations are commonly observed in solid tumors and are strongly associated with an increased tumor mutational burden as well as improved responses to immune checkpoint blockade therapy [9]. Shen et al. investigated the role of the long non-coding RNA TTN-AS1, which is related to TTN, and found that TTN-AS1 promotes endometrial cancer progression by sponging miR-376a-3p, thereby leading to the upregulation of PUM2, a gene involved in cell proliferation and migration [10]. Plectin, encoded by the PLEC gene, is a critical cytolinker protein that maintains cellular structural integrity by cross-linking cytoskeletal components, playing a pivotal role in tissue mechanics and mechanotransduction [11]. Perez et al. explored the function of PLEC in cancer development and progression, demonstrating that PLEC, through its roles in cancer cell proliferation, adhesion, migration, invasion, and signal transduction, contributes to tumorigenesis when mislocalized to the cell surface in cancer cells [12]. Additionally, proteases from the PRSS family, secreted by cancer cells, promote tumor progression by activating pro-urokinase and pro–matrix metalloproteinase, facilitating extracellular matrix degradation, angiogenesis, and tumor invasion [13]. Some studies have reported the upregulation of PRSS1 in ovarian cancer and its association with platinum resistance. Although these findings primarily pertain to ovarian cancer, they suggest that PRSS1 may be implicated in chemoresistance mechanisms relevant to endometrial cancer therapy as well [14].

Recent advancements in molecular oncology have led to a refined classification of endometrial cancer. In 2013, The Cancer Genome Atlas (TCGA) identified four molecular subtypes, providing deeper insights into tumor behavior and prognosis [8].

1. POLE-ultramutated subtype: Characterized by a high frequency of mutations due to alterations in the POLE gene, which encodes a subunit of DNA polymerase.
2. Microsatellite instability (MSI) hypermutated subtype: Defined by defects in DNA mismatch repair, leading to high mutational burden.
3. Copy-number low subtype: Associated with a relatively stable genome and endometrioid histology.
4. Copy-number high (serous-like) subtype: Marked by widespread genomic instability and frequent copy-number alterations.

Numerous studies in the existing literature have validated various clinical prediction models for endometrial cancer, with a growing trend towards integrating molecular and genetic markers alongside traditional clinicopathological features to enhance prediction accuracy and individualization. Notably, certain studies suggest that incorporating the molecular subtypes of endometrial cancer—such as POLE ultramutated and MMR-deficient variants—into clinical models can significantly improve prognostication, especially for advanced-stage disease [15].

In recent years, circulating cell-free DNA (ccfDNA) has gained attention as a potential biomarker for non-invasive cancer detection and monitoring. A fraction of ccfDNA, known as circulating tumor DNA (ctDNA), originates from tumor cells and carries mutations reflective of the underlying malignancy [16]. The application of liquid biopsy in oncology facilitates repeated sampling for tumor genomic profiling, enhancing diagnostic precision and therapeutic decision-making [17]. The emergence of next-generation sequencing (NGS) has further enabled the detection of clinically relevant mutations in plasma-derived ccfDNA, offering promising avenues for personalized medicine in endometrial cancer [18].

Transvaginal ultrasonography (TVUS) remains the standard non-invasive technique for the early detection and assessment of endometrial pathologies, including endometrial cancer. This imaging modality provides a reliable evaluation of endometrial thickness, which serves as an important screening criterion [19]. Additionally, shear wave elastography, an advanced ultrasound technique that quantifies tissue stiffness, has been explored for distinguishing malignant from benign endometrial lesions [20]. While preoperative histological tumor grading using probe curettage correlates with final pathological findings, it may not always provide definitive diagnostic accuracy. Clinical decisions should therefore integrate multiple diagnostic modalities [21].

Shear Wave Elastography (SWE) has demonstrated a statistically significant difference in tissue stiffness between malignant and benign endometrial pathologies. In patients presenting with abnormal uterine bleeding, mean elasticity (Emean) values were markedly higher in cases of endometrial cancer compared to benign lesions and normal endometrium. These findings suggest that SWE may serve as a valuable non-invasive diagnostic adjunct in differentiating endometrial malignancies from benign conditions by quantitatively assessing endometrial stiffness [22].

Despite these advancements, there remains a pressing need for more precise, less invasive, and cost-effective diagnostic tools for preoperative evaluation, surgical decision-making, and treatment planning in endometrial cancer. The present study aims to contribute to the refinement of preoperative diagnostic methodologies by integrating emerging molecular and imaging-based techniques.

## 2. Materials and Methods

The study included 47 patients diagnosed with endometrial cancer based on biopsy results, 29 of whom underwent shear wave elastography (SWE), which was conducted at Kocaeli University Hospital between 2022 and 2023. Patients from whom consent for elastography could not be obtained were excluded from the study. Ethics committee approval was obtained from Kocaeli University in 30 December 2021 (Clinical Research Ethics Committee no: 2021/23.14).

### 2.1. Study Design and Sample Collection

This study was conducted at Kocaeli University, Faculty of Medicine. Department of Obstetrics and Gynecology in collaboration with the Genetic Diseases Diagnosis Center. Patients who met the eligibility criteria were included in the study and their genetic sequencing was performed using NGS. All coding regions of the selected genes were analyzed. In addition, SWE imaging was performed preoperatively in the Radiology Department for participants.

### 2.2. Genetic Analysis

#### 2.2.1. DNA Isolation from Peripheral Blood

Peripheral blood samples were collected in EDTA tubes from all participants. DNA isolation was performed using an automated Qiagen robotic system, which utilizes a cartridge-based approach containing Proteinase K, washing solutions, magnetic beads, and pipetting wells. Following the automatized DNA isolation process, a total of 50 μL of DNA (containing 30–100 ng/μL) was obtained from 220 μL of peripheral blood.

#### 2.2.2. DNA Quality and Quantification

After DNA extraction genomic DNA quality and quantity were assessed using a NanoDrop spectrophotometer. A 2 μL DNA sample was analyzed spectrophotometrically and the results were recorded. DNA samples were diluted accordingly to meet the required concentrations.

#### 2.2.3. Qubit Fluorometric Quantification

Qubit™ dsDNA HS Assay Kit (Thermo Fisher Scientific, Waltham, MA, USA) was used following the manufacturer’s protocol to confirm DNA usability for NGS gene panel analysis. Before measurement all reagents were vortexed and a working solution (1:200 dilution) was prepared by mixing 1 μL reagent with 199 μL buffer for each DNA sample. Two microliters (2 μL) of DNA were added to 198 μL of the working solution and vortexed. Measurements were performed using the “dsDNA-High Sensitivity” mode. Based on the results. DNA concentrations were adjusted to 5 ng/μL using sterile water.

#### 2.2.4. NGS Analysis

The NGS-based genetic analysis was conducted using the Hereditary Cancer Panel which includes genes associated with hereditary cancer syndromes. The sequencing process was carried out on the DNBSEQ-G400 platform (MGI Tech Co., Ltd., Shenzhen, Guangdong, China).

#### 2.2.5. NGS Library Preparation and Sequencing

The sequencing library preparation was performed on the DNBSEQ-G400 system. Eight-sample sets of prepared libraries underwent concentration measurement and were then pooled in equal concentrations into a single tube following the instrument’s guidelines. The sequencing reactions were initiated after loading the pooled library into the NGS system.

#### 2.2.6. Bioinformatics Analysis of Sequencing Data

Post-sequencing the raw sequencing data underwent bioinformatics analysis using multiple databases, including ClinVar, dbSNP, MutationTaster, PresentSome, and Franklin to interpret the identified genetic variants (Table 1). The classification of variants followed the American College of Medical Genetics (ACMG) guidelines [23], (Table 2) categorizing them as pathogenic (P), likely pathogenic (LP) and variants of uncertain significance (VUS). Minor allele frequency (MAF) values of detected variants were retrieved from gnomAD (The Genome Aggregation Database) (Table 1). Variants not listed in ClinVar were classified according to ACMG criteria (Table 2).

### 2.3. Elastography Analysis

**Figure 1:**
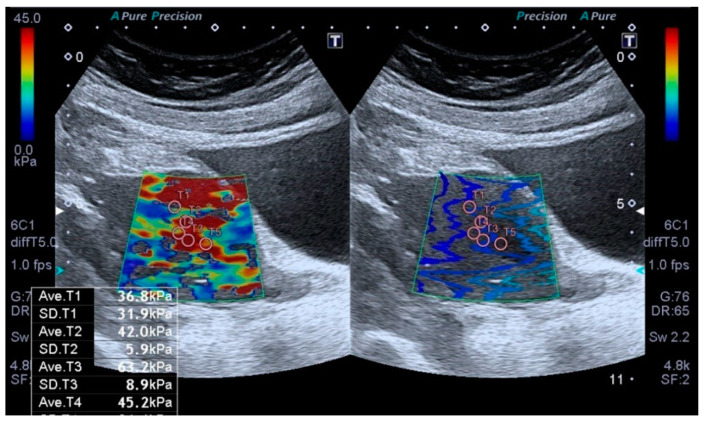
Transabdominal ultrasound examination of the endometrium, including shear wave elastography (SWE). Placement of sample points of the region of interest (ROI) in the endometrial lesion for SWE measurements.

All sonographic and elastographic examinations were performed by the same radiologist who has 12 years’ experience in genitourinary radiology. A digital sonography scanner (Aplio i500, Canon Medical Systems, Tustin, CA, USA, equipped with a 3–5 MHz convex probe) was used to perform real-time tissue elastography which is a shear wave-based technology. The uterus was scanned in the coronal and longitudinal projections. The endometrial thickness was measured by B-mode ultrasound in the sagittal plan. Elastography examinations were performed by holding the breath and lightly touching the probe skin to minimize movement artifacts. After obtaining conventional US images, the target area was determined, and measurements were taken from the region of interest (ROI) (Figure 1).

The region of interests (ROIs) were adjusted to include maximum homogeneous and thick tissue to avoid the ROI bias. The fixed ROI box size of our study was 1–0.5 cm. The elastographic images that exhibited color homogeneity were selected for elastographic data analysis. Five regions of interest were set for the SWE images, which were displayed as a translucent color map superimposed on the corresponding B-mode image. Tissue elasticity measurements were made using a map with a color range from dark blue to red (soft to hard). The stiffness value was obtained to evaluate the stiffness of the endometrium quantitatively. To evaluate endometrial stiffness, 5 valid measurements were taken from each patient’s endometrial lesion, and their average was calculated. The results were expressed in kPa. From the five measurements, the mean, and IQR/median values were calculated, and only measurements with IQR/median ratios ≤30% were considered acceptable. All US images were sent to the workstation and archived in the picture archiving and communication system (PACS) (Sectra IDS 7, Sectra, Linköping, Sweden). The average time to obtain a reliable measurement was about 20–30 min.

### 2.4. Statistical Analysis

All statistical analyses were conducted using NCSS 2020 Statistical Software 20.0.8 (NCSS LLC, Kaysville, UT, USA). Descriptive statistics were used to summarize the study data with quantitative variables presented as mean, standard deviation (SD), and median with minimum and maximum values while categorical variables were expressed as frequency and percentage. The Shapiro-Wilk test and Box Plot analysis were applied to assess the normality of data distribution. For comparisons between two independent groups with non-normally distributed variables the Mann-Whitney U test was utilized. Correlations between continuous variables were analyzed using Spearman’s correlation test. For the comparison of categorical variables Fisher’s Exact test and Fisher’s Freeman-Halton test were applied. Statistical significance was determined at a 95% confidence interval, with a *p*-value <0.05 considered statistically significant.

## 3. Results

The age range of the participants was 30–84 years with a mean age of 59.45 ± 10.70 years. The gravida was reported between 0 and 9, with a mean of 3.30 ± 2.09 (Table 3).

**Figure 2:**
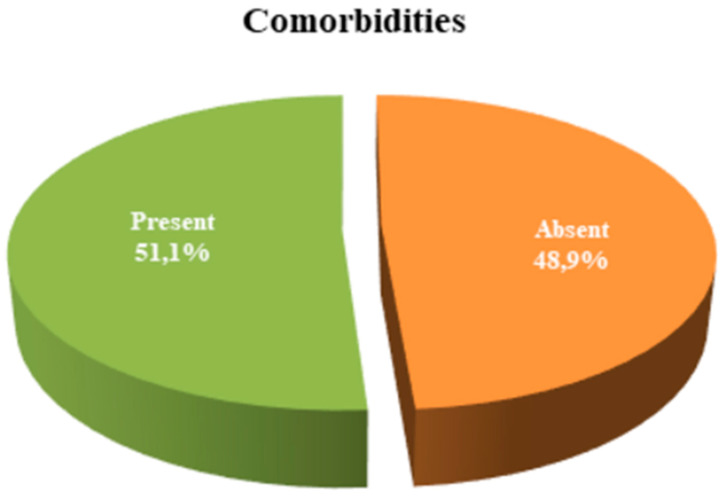
Distribution of presence and absence of comorbidities.

Comorbidities were present in 51.1% of the participants (n = 24) (Figure 2).

The USG measurements of endometrial thickness ranged from 5.5 to 47 mm, with a mean value of 19.93 ± 9.83 mm. The kPa values varied between 19.1 and 70.4, with a mean kPa of 39.56 ± 14.21. Among the participants, 62.1% (n = 18) had kPa <40, while 37.9% (n = 11) had kPa ≥40 (Table 4). The number of gene mutations ranged from 0 to 6, with an average of 2.28 ± 1.96 mutations per participant. Genetic mutations were detected in 66% (n = 31) of cases, with 22.6% (n = 7) having 1–2 mutations, 61.3% (n = 19) having 3–4 mutations, and 16.1% (n = 5) having 5–6 mutations (Table 4).

**Figure 3:**
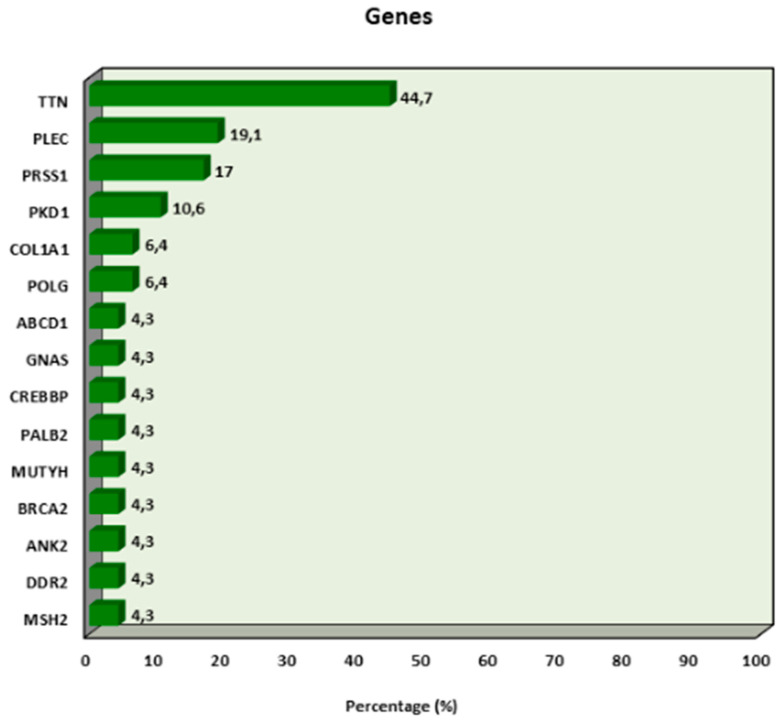
Distribution of genes in which mutations have been associated with endometrial cancer.

The genes present in the cases are as follows: TTN in 44.7% (n = 21), PLEC in 19.1% (n = 9), PRSS1 in 17% (n = 8), PKD1 in 10.6% (n = 5), COL1A1 in 6.4% (n = 3), and POLG in 6.4% (n = 3) (Figure 3).

The median kPa level was 33 in individuals with gene mutations, whereas it was 42.2 in those without detected mutations. However, this difference was not statistically significant (*p* > 0.05). No statistically significant differences were observed in KPA levels between individuals with and without mutations in the *TTN*, *PLEC*, *PRSS1*, *PKD1*, *COL1A1*, and *POLG* genes (*p* > 0.05). Similarly, when kPa levels were categorized based on a threshold of 40, no statistically significant difference was found between individuals with and without mutations in these genes (*p* > 0.05) (Table 5).

The presence of gene mutations, the number of mutations, and mutations in the *TTN*, *PLEC*, *PRSS1*, *PKD1*, *COL1A1*, and *POLG* genes were not statistically associated with metastasis (*p* > 0.05). The median kPA level was 40.1 in cases with metastasis, whereas it was 34.7 in those without (Table 6). Although kPA levels were higher in cases with metastasis, this difference was not statistically significant due to the limited sample size (*p* > 0.05). However, it is anticipated that larger studies may yield statistically significant results.

## 4. Discussion

Expanding clinical diagnostic techniques and refining the understanding of biological characteristics have been proposed as essential strategies for improving early cancer diagnosis and tailoring more individualized treatment approaches. Ultrasound-based elastography has been widely evaluated as a noninvasive tool for assessing tumor stiffness, with studies demonstrating high sensitivity and specificity in diagnosing breast tumors [24,25,26].

However, elastography has not been extensively investigated for differentiating the stiffness of endometrial carcinoma from benign endometrial pathologies. The present study aimed to evaluate the accuracy of elastography in detecting endometrial carcinoma and its potential associations with genetic mutations. Previous studies have shown that higher tumor stiffness in invasive breast cancer correlates with a higher histological grade and significantly poorer survival outcomes compared to tumors with lower stiffness [24,25,26].

Similarly, increased tissue stiffness in endometrial cancer compared to normal endometrial tissue has been demonstrated using strain elastography, with a significant difference in stiffness ratio observed in postmenopausal cases [27]. Consistent with these findings, our study identified a median kPa level of 40.1 in metastatic cases, compared to a median of 34.7 in non-metastatic cases. This suggests that metastasis and more aggressive forms of endometrial carcinoma are associated with stiffer tissue.

Furthermore, tumors with higher stiffness may harbor additional molecular mutations, contributing to tumor progression. While ultrasound elastography combined with shear wave elastography (UE-SWE) has been established as a useful tool for distinguishing benign from malignant breast lesions, its association with genetic alterations in fibroepithelial tumors remains under investigation [28,29]. The study of UE-SWE and its relevance to genetic alterations in fibroadenomas (FA) may provide insights into the molecular pathogenesis of fibroepithelial breast tumors and breast cancer. However, the primary genetic and molecular alterations associated with FA tumorigenesis remain unclear [28,29].

Recent studies suggest that TTN mutations are associated with poor overall survival outcomes in solid tumors while demonstrating a positive response to immune checkpoint inhibitors, underscoring the potential role of TTN in tumor immunogenicity [9]. The adverse impact of TTN mutations on solid tumor prognosis is largely attributed to its association with chemotherapy resistance [9].

While this study makes valuable contributions, it is not without limitations. As a primary limitation, the relatively small sample size may have restricted the detection of more genomic alterations. Nevertheless, recurrent mutations, including those in TTN, PLEC, and PRSS1, were identified in the cohort. Secondly, the study did not compare copy number variation landscapes between circulating tumor DNA (ctDNA) and matched tumor tissue, potentially affecting the reliability of genomic findings. Prior studies have demonstrated the concordance of mutation patterns between ctDNA and tumor tissue, supporting the potential of liquid biopsy approaches [30].

Type II endometrial cancers are characterized by non-endometrioid histology, with alterations in TP53 and P16, HER-2 overexpression, high-grade features, and loss of hormone receptor expression. In alignment with prior findings on breast cancer, patients with higher tumor stiffness exhibited a higher histological grade and significantly poorer survival compared to those with lower stiffness [24,25,26]. These observations further support the hypothesis that tumor stiffness may be associated with tumor aggressiveness and molecular alterations.

Despite its contributions, this study has several limitations. First, the relatively small sample size, especially the limited number of patients with metastatic disease, may have reduced the statistical power to detect associations between SWE values and specific gene mutations. Second, SWE was performed using a transabdominal convex probe rather than a transvaginal probe, due to equipment availability. Although abdominal SWE is a feasible alternative, it may be influenced by factors such as abdominal wall adiposity, and the potential impact of body mass index (BMI) on elastographic measurements was not systematically assessed in this study. Third, elastography examinations are operator-dependent despite standardization efforts, and minor technical variations may have introduced measurement variability. Fourth, the study focused primarily on cross-sectional diagnostic associations and did not incorporate longitudinal survival outcomes, such as cancer-specific mortality, which limits conclusions regarding the prognostic value of SWE findings or genetic mutations. Nevertheless, our study has several notable strengths. To our knowledge, this is among the first investigations to explore the relationship between endometrial tissue stiffness measured by SWE and genetic alterations detected by NGS in endometrial cancer. All elastographic examinations were performed by a single experienced radiologist, minimizing inter-operator variability. Moreover, the integration of imaging data with comprehensive genetic profiling broadens the scope of diagnostic research in endometrial cancer and provides a foundation for future studies aiming to personalize management strategies. Our findings highlight the potential role of SWE as a non-invasive adjunct in the preoperative assessment of endometrial cancer aggressiveness, warranting validation in larger, prospective cohorts.

In conclution, although our study did not establish a statistically significant relationship between gene mutations and kPa levels, the elevated tissue stiffness observed in metastatic cases reinforces the need for further large-scale investigations to validate our hypothesis. Elastography has emerged as a promising tool for differentiating between malignant and benign endometrial tissues. As a noninvasive imaging modality, elastography holds potential in facilitating the diagnosis of endometrial carcinoma without requiring invasive tissue sampling procedures.

## Figures and Tables

**Table 1:** Databases Used for Variant Interpretation.

Database	URL
ClinVar	https://www.ncbi.nlm.nih.gov/clinvar/ (accessed on 1 May 2021)
dbSNP	https://www.ncbi.nlm.nih.gov/snp/ (accessed on 1 May 2021)
MutationTaster	http://www.mutationtaster.org/ (accessed on 1 May 2021)
VarSome	https://varsome.com/ (accessed on 1 May 2021)
Franklin	https://franklin.genoox.com/clinical-db/home (accessed on 1 May 2021)
gnomAD	https://gnomad.broadinstitute.org/ (accessed on 1 May 2021)

**Table 2:** ACMG Classification Criteria.

Classification	Criteria and Evidence Codes
Pathogenic (P)	1 Very Strong (PVS1) AND ≥1 Strong (PS1–PS4) OR ≥2 Moderate (PM1–PM6) OR 1 Moderate (PM1–PM6) + 1 Supporting (PP1–PP5) OR ≥2 Supporting (PP1–PP5) ≥2 Strong (PS1–PS4) OR 1 Strong (PS1–PS4) AND ≥3 Moderate (PM1–PM6) OR 2 Moderate (PM1–PM6) + ≥2 Supporting (PP1–PP5) OR 1 Moderate (PM1–PM6) + ≥4 Supporting (PP1–PP5)
Likely Pathogenic (LP)	1 Very Strong (PVS1) + 1 Moderate (PM1–PM6) OR 1 Strong (PS1–PS4) + 1–2 Moderate (PM1–PM6) OR 1 Strong (PS1–PS4) + ≥2 Supporting (PP1–PP5) OR ≥3 Moderate (PM1–PM6) OR 2 Moderate (PM1–PM6) + ≥2 Supporting (PP1–PP5) OR 1 Moderate (PM1–PM6) + ≥4 Supporting (PP1–PP5)
Benign (B)	1 Independent (BA1) OR ≥2 Strong (BS1–BS4)
Likely Benign (LB)	1 Strong (BS1–BS4) + 1 Supporting (BP1–BP7) OR ≥2 Supporting (BP1–BP7)
Variants of Uncertain Significance (VUS)	Criteria for other categories are not met. OR Conflicting pathogenic and benign criteria

**Table 3:** Distribution of demographic characteristics.

Variable	Mean ± SD	Median (Min–Max)
Age (years)	59.45 ± 10.70	62 (30–84)
Gravida	3.30 ± 2.09	3 (0–9)
Parity	2.64 ± 1.74	3 (0–8)
Height (cm) (n = 31)	161.65 ± 5.10	162 (150–170)
Weight (cm) (n = 31)	94.06 ± 19.62	87 (65–139)
BMI (kg/m^2^) (n = 30)	36.11 ± 7.61	35 (25.5–57.9)
Comorbidities	n (%)	
Absent	23 (48.9)	
Present	24 (51.1)	
Medication Use	n (%)	
No	14 (29.8)	
Yes	33 (70.2)	
Diabetes Mellitus (DM)	n (%)	
No	35 (74.5)	
Yes	12 (25.5)	
Hypertension (HT)	n (%)	
No	23 (48.9)	

BMI: Body mass index.

**Table 4:** Distribution of hematological, biochemical, ultrasound and genetic parameters.

Parameters	Mean ± SD	Median (Min–Max)
HB (g/dL)	13.09 ± 1.68	13.5 (8.5–17.3)
HCT (%)	39.30 ± 4.50	39.6 (27.1–49.6)
WBC (×10^3^/µL)	8.36 ± 2.43	7.8 (4.8–17.8)
LYM (×10^3^/µL)	2.32 ± 0.89	2.3 (0.4–4.3)
EOS (%)	5.29 ± 2.34	4.8 (3.1–15.3)
PLT (×10^3^/µL)	277.16 ± 61.82	267 (155–418)
ALT (U/L)	21.23 ± 10.78	18 (6.4–54.6)
AST (U/L)	21.15 ± 7.91	19 (11.3–48.2)
LDH (U/L) (n = 41)	221.12 ± 50.66	216 (151–381)
Urea (mg/dL)	30.76 ± 9.59	29.1 (13.1–55.7)
Creatinine (mg/dL)	0.76 ± 0.16	0.8 (0.5–1.2)
Albumin (g/L) (n = 45)	43.07 ± 4.34	44.3 (26.5–50)
US Measurement (mm) (n = 38)	19.93 ± 9.83	16.5 (5.5–47)
**Treatment (n = 43)**	**n (%)**	
BT	24 (55.8%)	
BT + CT	2 (4.7%)	
Intensity-Modulated Radiation Therapy	1 (2.3%)	
Only CT	2 (4.7%)	
CT + RT	1 (2.3%)	
Follow-up Only	13 (30.2%)	
**Shear Wave Elastography (SWE. kPa) (n = 29)**	**Mean ± SD**	**Median (Min–Max)**
kPa Measurement	39.56 ± 14.21	36.5 (19.1–70.4)
<40 kPa n (%)	18 (62.1%)	
≥40 kPa n (%)	11 (37.9%)	
Number of Gene Mutations	2.28 ± 1.96	3 (0–6)
**Mutation Status**	**n (%)**	
Absent	16 (34.0%)	
Present	31 (66.0%)	
1–2 Mutations	7 (22.6%)	
3–4 Mutations	19 (61.3%)	
5–6 Mutations	5 (16.1%)	

HB: Hemoglobin, HCT: Hematocrit, WBC: White Blood Cells, LYM: Lymphocyte, EOS: Eosinophils, PLT: Platelets, ALT: Alanine Aminotransferase, AST: Aspartate Aminotransferase, LDH: Lactate Dehydrogenase, US: Ultrasound, BT: Brachytherapy, CT: Chemotherapy, RT: Radiotherapy.

**Table 5:** Comparison of gene mutations according to kPa values.

		kPa		^a^ *p*	kPa		^b^ *p*
		Mean ± SD	Median (Min–Max)		<40	≥40	
Gene mutation	Absent	42.99 ± 14.24	42.2 (25–63.9)	0.374	4 (22.2)	4 (36.4)	0.433
	Present	38.26 ± 14.32	33 (19.1–70.4)		14 (77.8)	7 (63.6)	
TTN	Absent	41.44 ± 15.69	36.5 (24.4–70.4)	0.650	10 (55.6)	6 (54.5)	1.000
	Present	37.26 ± 12.36	37.7 (19.1–63.6)		8 (44.4)	5 (45.5)	
PLEC	Absent	40.61 ± 14.91	36.5 (23.2–70.4)	0.618	14 (77.8)	9 (81.8)	1.000
	Present	35.56 ± 11.32	34.1 (19.1–50.4)		4 (22.2)	2 (18.2)	
PRSS1	Absent	37.92 ± 13.13	36.5 (19.1–66)	0.328	15 (83.3)	8 (72.7)	0.646
	Present	45.85 ± 17.64	40.1 (30.5–70.4)		3 (16.7)	3 (27.3)	
PKD1	Absent	39.50 ± 13.64	36.5 (23.2–70.4)	0.973	16 (88.9)	10 (90.9)	1.000
	Present	40.12 ± 22.37	37.7 (19.1–63.6)		2 (11.1)	1 (9.1)	
COL1A1	Absent	40.11 ± 14.13	36.5 (23.2–70.4)	0.493	17 (94.4)	10 (90.9)	1.000
	Present	32.13 ± 18.48	32.1 (19.1–45.2)		1 (5.6)	1 (9.1)	
POLG	Absent	39.80 ± 14.41	36.9 (19.1–70.4)	0.897	17 (94.4)	11 (100.0)	1.000
	Present	33.00 ± 0.00	33 (33–33)		1 (5.6)	0 (0.0)	

^a^ Mann-Whitney-U Test. ^b^ Fisher Exact Test.

**Table 6:** Comparison of genetic mutations and kPa values according to the presence of metastasis.

		Metastasis		*p*
		Absent	Present	
Gene mutation	Absent	14 (32.6)	2 (50.0)	^b^ 0.597
	Present	29 (67.4)	2 (50.0)	
Mutation number	Mean ± SD	2.35 ± 1.97	1.50 ± 1.91	^a^ 0.449
	Median (Min–Max)	3 (0–6)	1 (0–4)	
TTN	Absent	23 (53.5)	3 (75.0)	^b^ 0.617
	Present	20 (46.5)	1 (25.0)	
PLEC	Absent	34 (79.1)	4 (100.0)	^b^ 0.574
	Present	9 (20.9)	0 (0.0)	
PRSS1	Absent	35 (81.4)	4 (100.0)	^b^ 1.000
	Present	8 (18.6)	0 (0.0)	
PKD1	Absent	38 (88.4)	4 (100.0)	^b^ 1.000
	Present	5 (11.6)	0 (0.0)	
COL1A1	Absent	40 (93.0)	4 (100.0)	^b^ 1.000
	Present	3 (7.0)	0 (0.0)	
POLG	Absent	40 (93.0)	4 (100.0)	^b^ 1.000
	Present	3 (7.0)	0 (0.0)	
kPa	Mean ± SD	38.64 ± 14.03	47.53 ± 16.09	^a^ 0.251
	Median (Min–Max)	34.7 (19.1–70.4)	40.1 (36.5–66)	

^a^ Mann-Whitney-U Test. ^b^ Fisher Exact Test.

## Data Availability

The datasets analyzed in this study are available from the corresponding author upon reasonable request and with permission of the local Ethics Committee.
